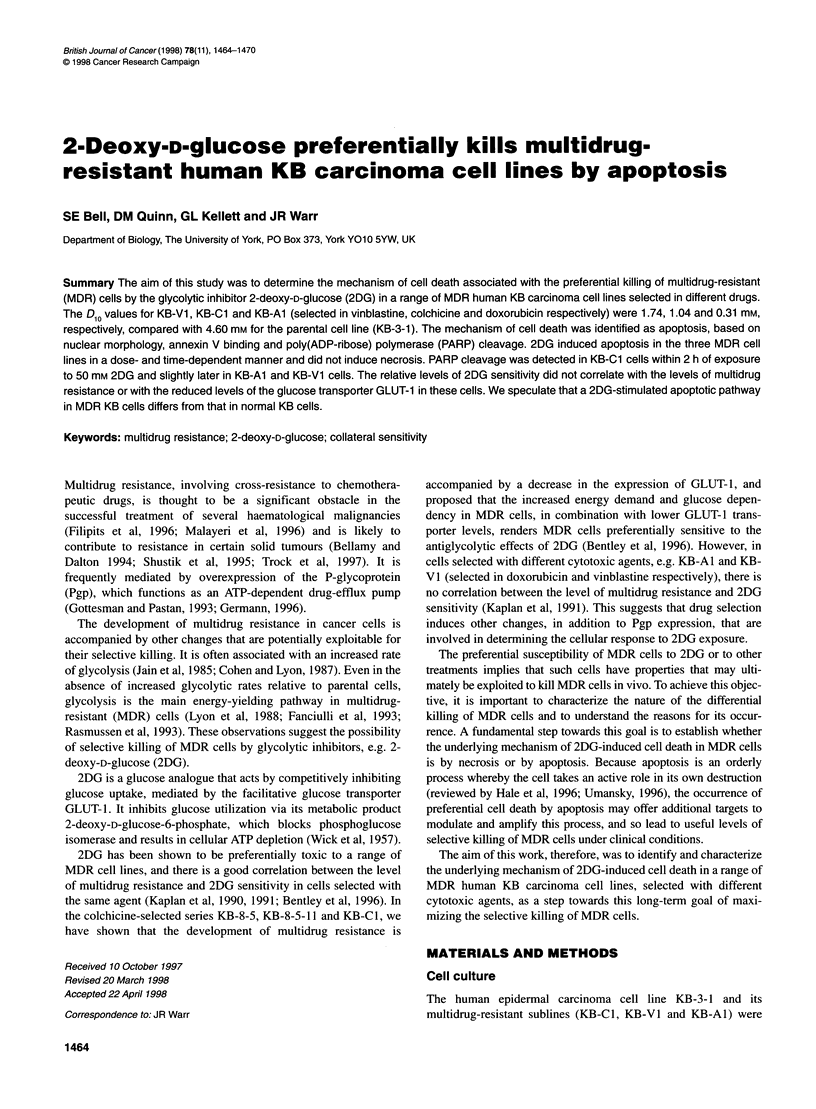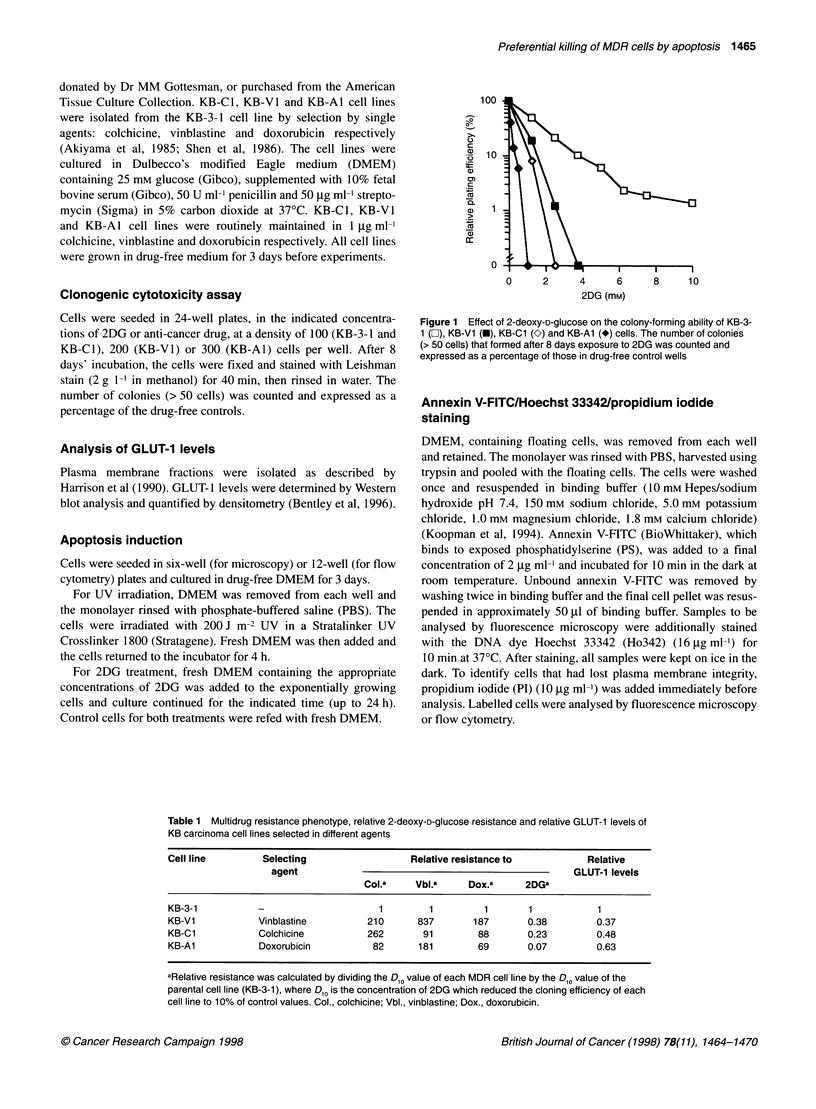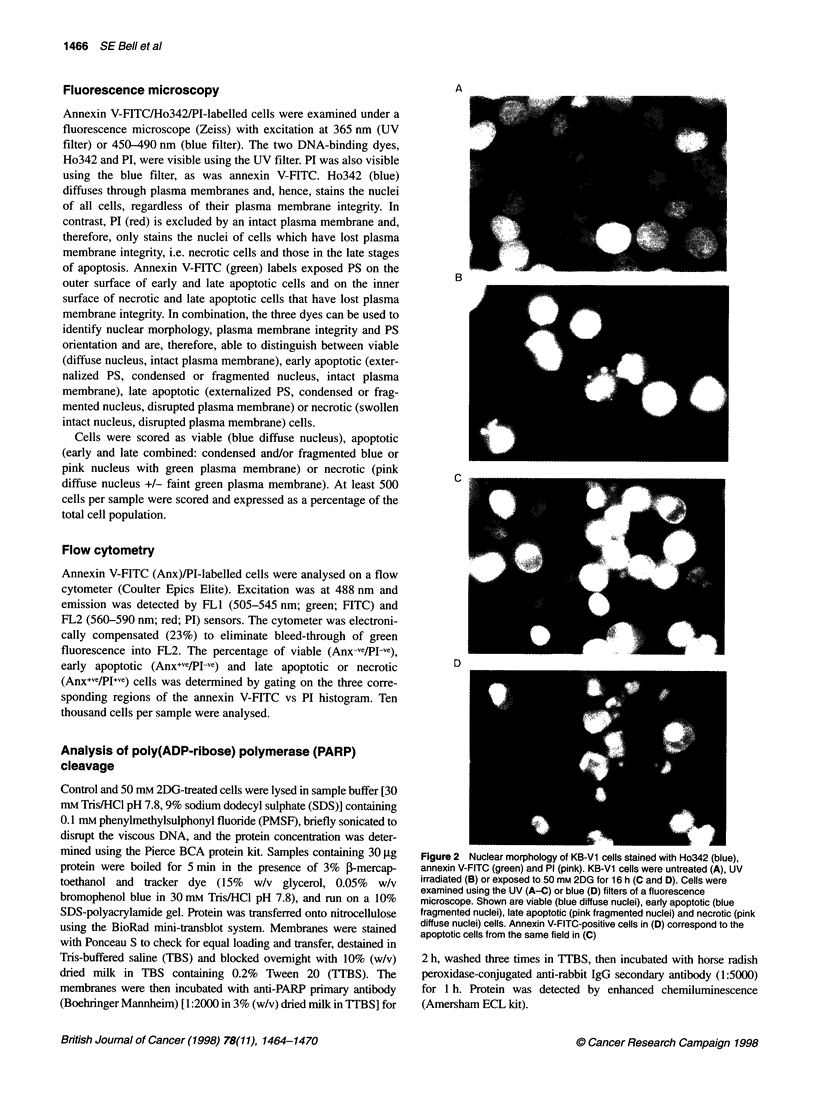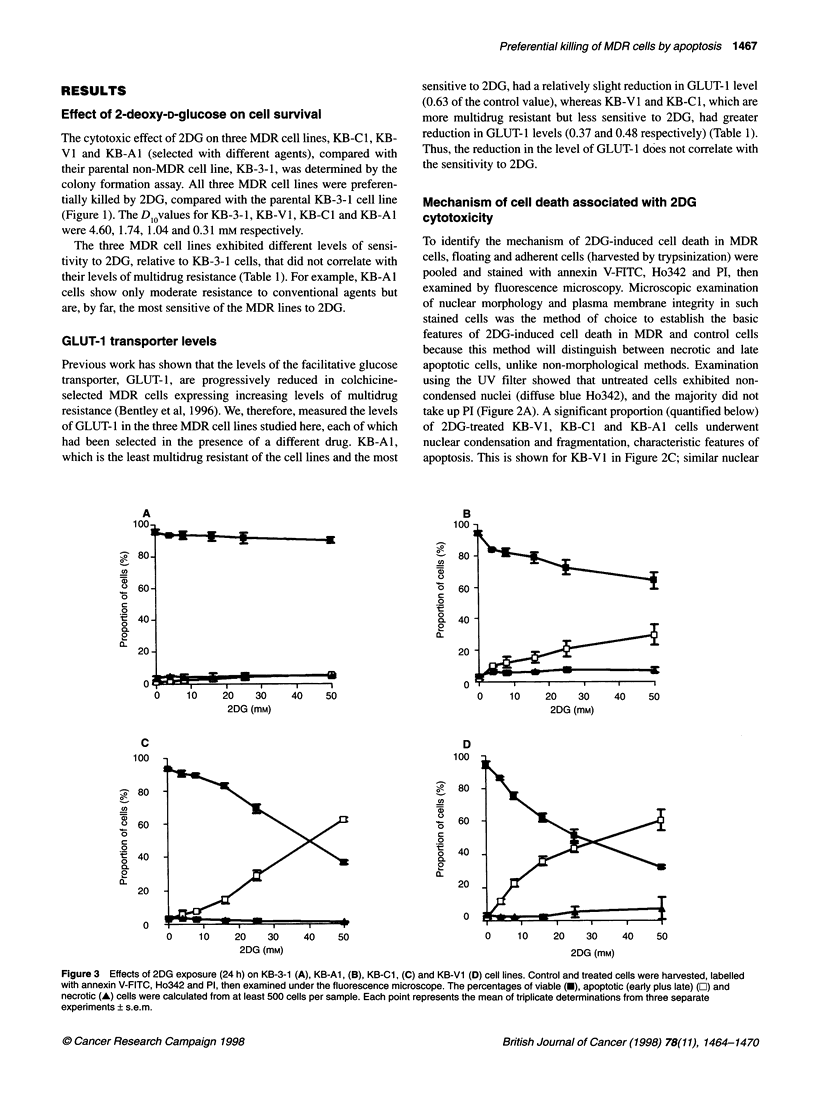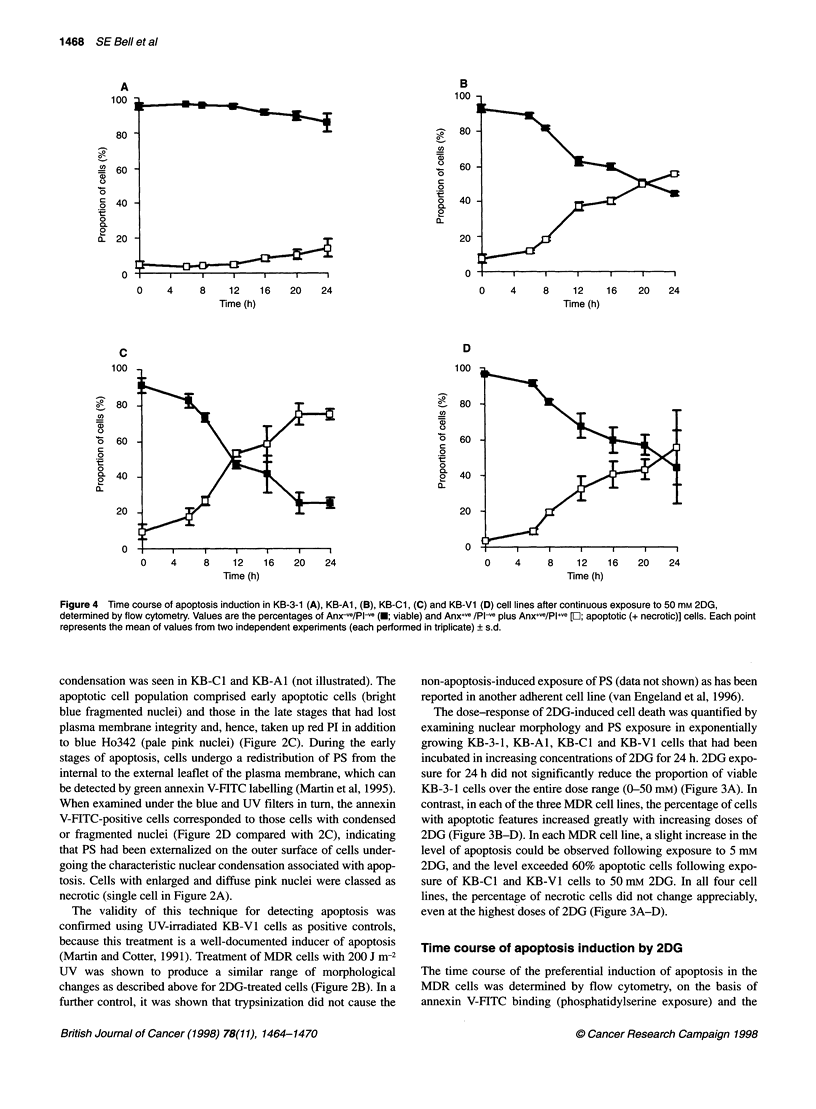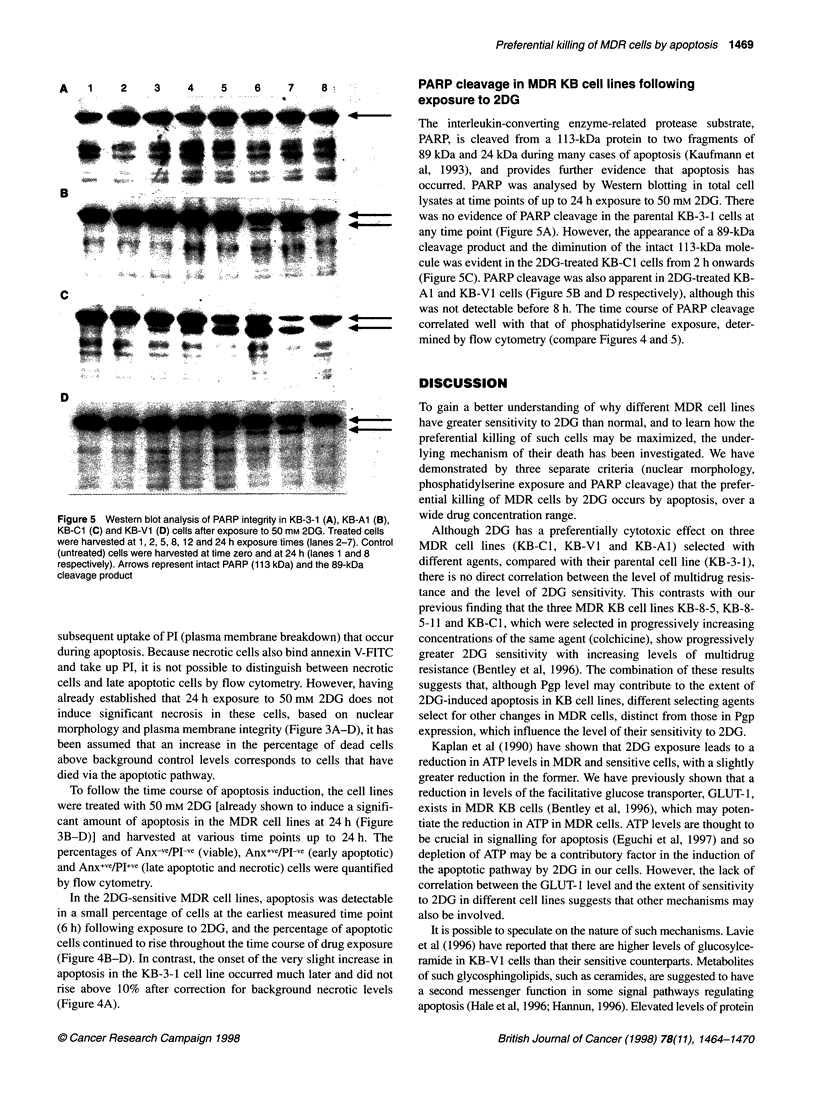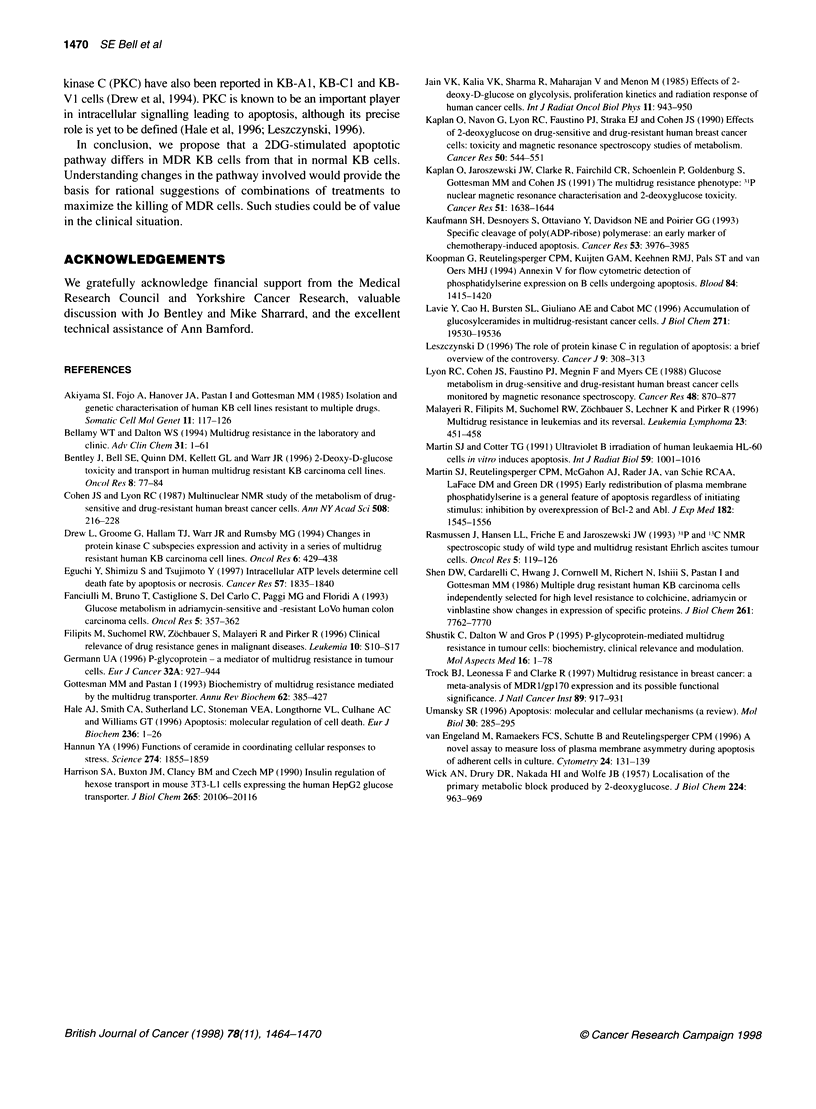# 2-Deoxy-D-glucose preferentially kills multidrug-resistant human KB carcinoma cell lines by apoptosis.

**DOI:** 10.1038/bjc.1998.708

**Published:** 1998-12

**Authors:** S. E. Bell, D. M. Quinn, G. L. Kellett, J. R. Warr

**Affiliations:** Department of Biology, The University of York, UK.

## Abstract

**Images:**


British Journal of Cancer (1998) 78(11), 1464–1470
© 1998 Cancer Research Campaign

### 2-Deoxy-D-glucose preferentially kills multidrug-resistant human KB carcinoma cell lines by apoptosis

**SE Bell, DM Quinn, GL Kellett and JR Warr**

Department of Biology, The University of York, PO Box 373, York YO10 5YW, UK

**Summary** The aim of this study was to determine the mechanism of cell death associated with the preferential killing of multidrug-resistant (MDR) cells by the glycolytic inhibitor 2-deoxy-D-glucose (2DG) in a range of MDR human KB carcinoma cell lines selected in different drugs. The $D_{10}$ values for KB-V1, KB-C1 and KB-A1 (selected in vinblastine, colchicine and doxorubicin respectively) were 1.74, 1.04 and 0.31 mM, respectively, compared with 4.60 mM for the parental cell line (KB-3-1). The mechanism of cell death was identified as apoptosis, based on nuclear morphology, annexin V binding and poly(ADP-ribose) polymerase (PARP) cleavage. 2DG induced apoptosis in the three MDR cell lines in a dose- and time-dependent manner and did not induce necrosis. PARP cleavage was detected in KB-C1 cells within 2 h of exposure to 50 mM 2DG and slightly later in KB-A1 and KB-V1 cells. The relative levels of 2DG sensitivity did not correlate with the levels of multidrug resistance or with the reduced levels of the glucose transporter GLUT-1 in these cells. We speculate that a 2DG-stimulated apoptotic pathway in MDR KB cells differs from that in normal KB cells.

**Keywords:** multidrug resistance; 2-deoxy-D-glucose; collateral sensitivity

Multidrug resistance, involving cross-resistance to chemotherapeutic drugs, is thought to be a significant obstacle in the successful treatment of several haematological malignancies (Filipits et al, 1996; Malayeri et al, 1996) and is likely to contribute to resistance in certain solid tumours (Bellamy and Dalton 1994; Shustik et al, 1995; Trock et al, 1997). It is frequently mediated by overexpression of the P-glycoprotein (Pgp), which functions as an ATP-dependent drug-efflux pump (Gottesman and Pastan, 1993; Germann, 1996).

The development of multidrug resistance in cancer cells is accompanied by other changes that are potentially exploitable for their selective killing. It is often associated with an increased rate of glycolysis (Jain et al, 1985; Cohen and Lyon, 1987). Even in the absence of increased glycolytic rates relative to parental cells, glycolysis is the main energy-yielding pathway in multidrug-resistant (MDR) cells (Lyon et al, 1988; Fanciulli et al, 1993; Rasmussen et al, 1993). These observations suggest the possibility of selective killing of MDR cells by glycolytic inhibitors, e.g. 2-deoxy-D-glucose (2DG).

2DG is a glucose analogue that acts by competitively inhibiting glucose uptake, mediated by the facilitative glucose transporter GLUT-1. It inhibits glucose utilization via its metabolic product 2-deoxy-D-glucose-6-phosphate, which blocks phosphoglucose isomerase and results in cellular ATP depletion (Wick et al, 1957).

2DG has been shown to be preferentially toxic to a range of MDR cell lines, and there is a good correlation between the level of multidrug resistance and 2DG sensitivity in cells selected with the same agent (Kaplan et al, 1990, 1991; Bentley et al, 1996). In the colchicine-selected series KB-8-5, KB-8-5-11 and KB-C1, we have shown that the development of multidrug resistance is accompanied by a decrease in the expression of GLUT-1, and proposed that the increased energy demand and glucose dependency in MDR cells, in combination with lower GLUT-1 transporter levels, renders MDR cells preferentially sensitive to the antiglycolytic effects of 2DG (Bentley et al, 1996). However, in cells selected with different cytotoxic agents, e.g. KB-A1 and KB-V1 (selected in doxorubicin and vinblastine respectively), there is no correlation between the level of multidrug resistance and 2DG sensitivity (Kaplan et al, 1991). This suggests that drug selection induces other changes, in addition to Pgp expression, that are involved in determining the cellular response to 2DG exposure.

The preferential susceptibility of MDR cells to 2DG or to other treatments implies that such cells have properties that may ultimately be exploited to kill MDR cells in vivo. To achieve this objective, it is important to characterize the nature of the differential killing of MDR cells and to understand the reasons for its occurrence. A fundamental step towards this goal is to establish whether the underlying mechanism of 2DG-induced cell death in MDR cells is by necrosis or by apoptosis. Because apoptosis is an orderly process whereby the cell takes an active role in its own destruction (reviewed by Hale et al, 1996; Umansky, 1996), the occurrence of preferential cell death by apoptosis may offer additional targets to modulate and amplify this process, and so lead to useful levels of selective killing of MDR cells under clinical conditions.

The aim of this work, therefore, was to identify and characterize the underlying mechanism of 2DG-induced cell death in a range of MDR human KB carcinoma cell lines, selected with different cytotoxic agents, as a step towards this long-term goal of maximizing the selective killing of MDR cells.

### MATERIALS AND METHODS

### Cell culture

The human epidermal carcinoma cell line KB-3-1 and its multidrug-resistant sublines (KB-C1, KB-V1 and KB-A1) were

*Received 10 October 1997*
*Revised 20 March 1998*
*Accepted 22 April 1998*

*Correspondence to:* JR Warr donated by Dr MM Gottesman, or purchased from the American Tissue Culture Collection. KB-C1, KB-V1 and KB-A1 cell lines were isolated from the KB-3-1 cell line by selection by single agents: colchicine, vinblastine and doxorubicin respectively (Akiyama et al, 1985; Shen et al, 1986). The cell lines were cultured in Dulbecco's modified Eagle medium (DMEM) containing 25 mM glucose (Gibco), supplemented with 10% fetal bovine serum (Gibco), 50 U $ml^{-1}$ penicillin and 50 µg $ml^{-1}$ streptomycin (Sigma) in 5% carbon dioxide at 37°C. KB-C1, KB-V1 and KB-A1 cell lines were routinely maintained in 1 µg $ml^{-1}$ colchicine, vinblastine and doxorubicin respectively. All cell lines were grown in drug-free medium for 3 days before experiments.

### Clonogenic cytotoxicity assay

Cells were seeded in 24-well plates, in the indicated concentrations of 2DG or anti-cancer drug, at a density of 100 (KB-3-1 and KB-C1), 200 (KB-V1) or 300 (KB-A1) cells per well. After 8 days' incubation, the cells were fixed and stained with Leishman stain (2 g $l^{-1}$ in methanol) for 40 min, then rinsed in water. The number of colonies (> 50 cells) was counted and expressed as a percentage of the drug-free controls.

### Analysis of GLUT-1 levels

Plasma membrane fractions were isolated as described by Harrison et al (1990). GLUT-1 levels were determined by Western blot analysis and quantified by densitometry (Bentley et al, 1996).

### Apoptosis induction

Cells were seeded in six-well (for microscopy) or 12-well (for flow cytometry) plates and cultured in drug-free DMEM for 3 days.

For UV irradiation, DMEM was removed from each well and the monolayer rinsed with phosphate-buffered saline (PBS). The cells were irradiated with 200 J $m^{-2}$ UV in a Stratalinker UV Crosslinker 1800 (Stratagene). Fresh DMEM was then added and the cells returned to the incubator for 4 h.

For 2DG treatment, fresh DMEM containing the appropriate concentrations of 2DG was added to the exponentially growing cells and culture continued for the indicated time (up to 24 h). Control cells for both treatments were refed with fresh DMEM.

**Figure 1** Effect of 2-deoxy-D-glucose on the colony-forming ability of KB-3-1 (□), KB-V1 (■), KB-C1 (◇) and KB-A1 (◆) cells. The number of colonies (> 50 cells) that formed after 8 days exposure to 2DG was counted and expressed as a percentage of those in drug-free control wells

### Annexin V-FITC/Hoechst 33342/propidium iodide staining

DMEM, containing floating cells, was removed from each well and retained. The monolayer was rinsed with PBS, harvested using trypsin and pooled with the floating cells. The cells were washed once and resuspended in binding buffer (10 mM Hepes/sodium hydroxide pH 7.4, 150 mM sodium chloride, 5.0 mM potassium chloride, 1.0 mM magnesium chloride, 1.8 mM calcium chloride) (Koopman et al, 1994). Annexin V-FITC (BioWhittaker), which binds to exposed phosphatidylserine (PS), was added to a final concentration of 2 µg $ml^{-1}$ and incubated for 10 min in the dark at room temperature. Unbound annexin V-FITC was removed by washing twice in binding buffer and the final cell pellet was resuspended in approximately 50 µl of binding buffer. Samples to be analysed by fluorescence microscopy were additionally stained with the DNA dye Hoechst 33342 (Ho342) (16 µg $ml^{-1}$) for 10 min at 37°C. After staining, all samples were kept on ice in the dark. To identify cells that had lost plasma membrane integrity, propidium iodide (PI) (10 µg $ml^{-1}$) was added immediately before analysis. Labelled cells were analysed by fluorescence microscopy or flow cytometry.

**Table 1** Multidrug resistance phenotype, relative 2-deoxy-D-glucose resistance and relative GLUT-1 levels of KB carcinoma cell lines selected in different agents

| Cell line | Selecting agent | Relative resistance to | | | | Relative GLUT-1 levels |
|---|---|---|---|---|---|---|
| | | Col.[a] | Vbl.[a] | Dox.[a] | 2DG[a] | |
| KB-3-1 | – | 1 | 1 | 1 | 1 | 1 |
| KB-V1 | Vinblastine | 210 | 837 | 187 | 0.38 | 0.37 |
| KB-C1 | Colchicine | 262 | 91 | 88 | 0.23 | 0.48 |
| KB-A1 | Doxorubicin | 82 | 181 | 69 | 0.07 | 0.63 |

[a]Relative resistance was calculated by dividing the $D_{10}$ value of each MDR cell line by the $D_{10}$ value of the parental cell line (KB-3-1), where $D_{10}$ is the concentration of 2DG which reduced the cloning efficiency of each cell line to 10% of control values. Col., colchicine; Vbl., vinblastine; Dox., doxorubicin.

© Cancer Research Campaign 1998

### Fluorescence microscopy

Annexin V-FITC/Ho342/PI-labelled cells were examined under a fluorescence microscope (Zeiss) with excitation at 365 nm (UV filter) or 450–490 nm (blue filter). The two DNA-binding dyes, Ho342 and PI, were visible using the UV filter. PI was also visible using the blue filter, as was annexin V-FITC. Ho342 (blue) diffuses through plasma membranes and, hence, stains the nuclei of all cells, regardless of their plasma membrane integrity. In contrast, PI (red) is excluded by an intact plasma membrane and, therefore, only stains the nuclei of cells which have lost plasma membrane integrity, i.e. necrotic cells and those in the late stages of apoptosis. Annexin V-FITC (green) labels exposed PS on the outer surface of early and late apoptotic cells and on the inner surface of necrotic and late apoptotic cells that have lost plasma membrane integrity. In combination, the three dyes can be used to identify nuclear morphology, plasma membrane integrity and PS orientation and are, therefore, able to distinguish between viable (diffuse nucleus, intact plasma membrane), early apoptotic (externalized PS, condensed or fragmented nucleus, intact plasma membrane), late apoptotic (externalized PS, condensed or fragmented nucleus, disrupted plasma membrane) or necrotic (swollen intact nucleus, disrupted plasma membrane) cells.

Cells were scored as viable (blue diffuse nucleus), apoptotic (early and late combined: condensed and/or fragmented blue or pink nucleus with green plasma membrane) or necrotic (pink diffuse nucleus +/– faint green plasma membrane). At least 500 cells per sample were scored and expressed as a percentage of the total cell population.

### Flow cytometry

Annexin V-FITC (Anx)/PI-labelled cells were analysed on a flow cytometer (Coulter Epics Elite). Excitation was at 488 nm and emission was detected by FL1 (505–545 nm; green; FITC) and FL2 (560–590 nm; red; PI) sensors. The cytometer was electronically compensated (23%) to eliminate bleed-through of green fluorescence into FL2. The percentage of viable ($Anx^{-ve}/PI^{-ve}$), early apoptotic ($Anx^{+ve}/PI^{-ve}$) and late apoptotic or necrotic ($Anx^{+ve}/PI^{+ve}$) cells was determined by gating on the three corresponding regions of the annexin V-FITC vs PI histogram. Ten thousand cells per sample were analysed.

### Analysis of poly(ADP-ribose) polymerase (PARP) cleavage

Control and 50 mM 2DG-treated cells were lysed in sample buffer [30 mM Tris/HCl pH 7.8, 9% sodium dodecyl sulphate (SDS)] containing 0.1 mM phenylmethylsulphonyl fluoride (PMSF), briefly sonicated to disrupt the viscous DNA, and the protein concentration was determined using the Pierce BCA protein kit. Samples containing 30 μg protein were boiled for 5 min in the presence of 3% β-mercaptoethanol and tracker dye (15% w/v glycerol, 0.05% w/v bromophenol blue in 30 mM Tris/HCl pH 7.8), and run on a 10% SDS-polyacrylamide gel. Protein was transferred onto nitrocellulose using the BioRad mini-transblot system. Membranes were stained with Ponceau S to check for equal loading and transfer, destained in Tris-buffered saline (TBS) and blocked overnight with 10% (w/v) dried milk in TBS containing 0.2% Tween 20 (TTBS). The membranes were then incubated with anti-PARP primary antibody (Boehringer Mannheim) [1:2000 in 3% (w/v) dried milk in TTBS] for 2 h, washed three times in TTBS, then incubated with horse radish peroxidase-conjugated anti-rabbit IgG secondary antibody (1:5000) for 1 h. Protein was detected by enhanced chemiluminescence (Amersham ECL kit).

**Figure 2** Nuclear morphology of KB-V1 cells stained with Ho342 (blue), annexin V-FITC (green) and PI (pink). KB-V1 cells were untreated (**A**), UV irradiated (**B**) or exposed to 50 mM 2DG for 16 h (**C** and **D**). Cells were examined using the UV (**A–C**) or blue (**D**) filters of a fluorescence microscope. Shown are viable (blue diffuse nuclei), early apoptotic (blue fragmented nuclei), late apoptotic (pink fragmented nuclei) and necrotic (pink diffuse nuclei) cells. Annexin V-FITC-positive cells in (**D**) correspond to the apoptotic cells from the same field in (**C**)

© Cancer Research Campaign 1998

### RESULTS

### Effect of 2-deoxy-D-glucose on cell survival

The cytotoxic effect of 2DG on three MDR cell lines, KB-C1, KB-V1 and KB-A1 (selected with different agents), compared with their parental non-MDR cell line, KB-3-1, was determined by the colony formation assay. All three MDR cell lines were preferentially killed by 2DG, compared with the parental KB-3-1 cell line (Figure 1). The $D_{10}$values for KB-3-1, KB-V1, KB-C1 and KB-A1 were 4.60, 1.74, 1.04 and 0.31 mM respectively.

The three MDR cell lines exhibited different levels of sensitivity to 2DG, relative to KB-3-1 cells, that did not correlate with their levels of multidrug resistance (Table 1). For example, KB-A1 cells show only moderate resistance to conventional agents but are, by far, the most sensitive of the MDR lines to 2DG.

### GLUT-1 transporter levels

Previous work has shown that the levels of the facilitative glucose transporter, GLUT-1, are progressively reduced in colchicine-selected MDR cells expressing increasing levels of multidrug resistance (Bentley et al, 1996). We, therefore, measured the levels of GLUT-1 in the three MDR cell lines studied here, each of which had been selected in the presence of a different drug. KB-A1, which is the least multidrug resistant of the cell lines and the most sensitive to 2DG, had a relatively slight reduction in GLUT-1 level (0.63 of the control value), whereas KB-V1 and KB-C1, which are more multidrug resistant but less sensitive to 2DG, had greater reduction in GLUT-1 levels (0.37 and 0.48 respectively) (Table 1). Thus, the reduction in the level of GLUT-1 does not correlate with the sensitivity to 2DG.

### Mechanism of cell death associated with 2DG cytotoxicity

To identify the mechanism of 2DG-induced cell death in MDR cells, floating and adherent cells (harvested by trypsinization) were pooled and stained with annexin V-FITC, Ho342 and PI, then examined by fluorescence microscopy. Microscopic examination of nuclear morphology and plasma membrane integrity in such stained cells was the method of choice to establish the basic features of 2DG-induced cell death in MDR and control cells because this method will distinguish between necrotic and late apoptotic cells, unlike non-morphological methods. Examination using the UV filter showed that untreated cells exhibited non-condensed nuclei (diffuse blue Ho342), and the majority did not take up PI (Figure 2A). A significant proportion (quantified below) of 2DG-treated KB-V1, KB-C1 and KB-A1 cells underwent nuclear condensation and fragmentation, characteristic features of apoptosis. This is shown for KB-V1 in Figure 2C; similar nuclear

**Figure 3** Effects of 2DG exposure (24 h) on KB-3-1 (**A**), KB-A1, (**B**), KB-C1, (**C**) and KB-V1 (**D**) cell lines. Control and treated cells were harvested, labelled with annexin V-FITC, Ho342 and PI, then examined under the fluorescence microscope. The percentages of viable (■), apoptotic (early plus late) (□) and necrotic (▲) cells were calculated from at least 500 cells per sample. Each point represents the mean of triplicate determinations from three separate experiments ± s.e.m.

**Figure 4** Time course of apoptosis induction in KB-3-1 (**A**), KB-A1, (**B**), KB-C1, (**C**) and KB-V1 (**D**) cell lines after continuous exposure to 50 mM 2DG, determined by flow cytometry. Values are the percentages of Anx$^{-ve}$/PI$^{-ve}$ (■; viable) and Anx$^{+ve}$ /PI$^{-ve}$ plus Anx$^{+ve}$/PI$^{+ve}$ [□; apoptotic (+ necrotic)] cells. Each point represents the mean of values from two independent experiments (each performed in triplicate) ± s.d.

condensation was seen in KB-C1 and KB-A1 (not illustrated). The apoptotic cell population comprised early apoptotic cells (bright blue fragmented nuclei) and those in the late stages that had lost plasma membrane integrity and, hence, taken up red PI in addition to blue Ho342 (pale pink nuclei) (Figure 2C). During the early stages of apoptosis, cells undergo a redistribution of PS from the internal to the external leaflet of the plasma membrane, which can be detected by green annexin V-FITC labelling (Martin et al, 1995). When examined under the blue and UV filters in turn, the annexin V-FITC-positive cells corresponded to those cells with condensed or fragmented nuclei (Figure 2D compared with 2C), indicating that PS had been externalized on the outer surface of cells undergoing the characteristic nuclear condensation associated with apoptosis. Cells with enlarged and diffuse pink nuclei were classed as necrotic (single cell in Figure 2A).

The validity of this technique for detecting apoptosis was confirmed using UV-irradiated KB-V1 cells as positive controls, because this treatment is a well-documented inducer of apoptosis (Martin and Cotter, 1991). Treatment of MDR cells with 200 J $m^{-2}$ UV was shown to produce a similar range of morphological changes as described above for 2DG-treated cells (Figure 2B). In a further control, it was shown that trypsinization did not cause the non-apoptosis-induced exposure of PS (data not shown) as has been reported in another adherent cell line (van Engeland et al, 1996).

The dose–response of 2DG-induced cell death was quantified by examining nuclear morphology and PS exposure in exponentially growing KB-3-1, KB-A1, KB-C1 and KB-V1 cells that had been incubated in increasing concentrations of 2DG for 24 h. 2DG exposure for 24 h did not significantly reduce the proportion of viable KB-3-1 cells over the entire dose range (0–50 mM) (Figure 3A). In contrast, in each of the three MDR cell lines, the percentage of cells with apoptotic features increased greatly with increasing doses of 2DG (Figure 3B–D). In each MDR cell line, a slight increase in the level of apoptosis could be observed following exposure to 5 mM 2DG, and the level exceeded 60% apoptotic cells following exposure of KB-C1 and KB-V1 cells to 50 mM 2DG. In all four cell lines, the percentage of necrotic cells did not change appreciably, even at the highest doses of 2DG (Figure 3A–D).

### Time course of apoptosis induction by 2DG

The time course of the preferential induction of apoptosis in the MDR cells was determined by flow cytometry, on the basis of annexin V-FITC binding (phosphatidylserine exposure) and the

© Cancer Research Campaign 1998

**Figure 5** Western blot analysis of PARP integrity in KB-3-1 (**A**), KB-A1 (**B**), KB-C1 (**C**) and KB-V1 (**D**) cells after exposure to 50 mM 2DG. Treated cells were harvested at 1, 2, 5, 8, 12 and 24 h exposure times (lanes 2–7). Control (untreated) cells were harvested at time zero and at 24 h (lanes 1 and 8 respectively). Arrows represent intact PARP (113 kDa) and the 89-kDa cleavage product subsequent uptake of PI (plasma membrane breakdown) that occur during apoptosis. Because necrotic cells also bind annexin V-FITC and take up PI, it is not possible to distinguish between necrotic cells and late apoptotic cells by flow cytometry. However, having already established that 24 h exposure to 50 mM 2DG does not induce significant necrosis in these cells, based on nuclear morphology and plasma membrane integrity (Figure 3A–D), it has been assumed that an increase in the percentage of dead cells above background control levels corresponds to cells that have died via the apoptotic pathway.

To follow the time course of apoptosis induction, the cell lines were treated with 50 mM 2DG [already shown to induce a significant amount of apoptosis in the MDR cell lines at 24 h (Figure 3B–D)] and harvested at various time points up to 24 h. The percentages of Anx$^{-ve}$/PI$^{-ve}$ (viable), Anx$^{+ve}$/PI$^{-ve}$ (early apoptotic) and Anx$^{+ve}$/PI$^{+ve}$ (late apoptotic and necrotic) cells were quantified by flow cytometry.

In the 2DG-sensitive MDR cell lines, apoptosis was detectable in a small percentage of cells at the earliest measured time point (6 h) following exposure to 2DG, and the percentage of apoptotic cells continued to rise throughout the time course of drug exposure (Figure 4B–D). In contrast, the onset of the very slight increase in apoptosis in the KB-3-1 cell line occurred much later and did not rise above 10% after correction for background necrotic levels (Figure 4A).

### PARP cleavage in MDR KB cell lines following exposure to 2DG

The interleukin-converting enzyme-related protease substrate, PARP, is cleaved from a 113-kDa protein to two fragments of 89 kDa and 24 kDa during many cases of apoptosis (Kaufmann et al, 1993), and provides further evidence that apoptosis has occurred. PARP was analysed by Western blotting in total cell lysates at time points of up to 24 h exposure to 50 mM 2DG. There was no evidence of PARP cleavage in the parental KB-3-1 cells at any time point (Figure 5A). However, the appearance of a 89-kDa cleavage product and the diminution of the intact 113-kDa molecule was evident in the 2DG-treated KB-C1 cells from 2 h onwards (Figure 5C). PARP cleavage was also apparent in 2DG-treated KB-A1 and KB-V1 cells (Figure 5B and D respectively), although this was not detectable before 8 h. The time course of PARP cleavage correlated well with that of phosphatidylserine exposure, determined by flow cytometry (compare Figures 4 and 5).

### DISCUSSION

To gain a better understanding of why different MDR cell lines have greater sensitivity to 2DG than normal, and to learn how the preferential killing of such cells may be maximized, the underlying mechanism of their death has been investigated. We have demonstrated by three separate criteria (nuclear morphology, phosphatidylserine exposure and PARP cleavage) that the preferential killing of MDR cells by 2DG occurs by apoptosis, over a wide drug concentration range.

Although 2DG has a preferentially cytotoxic effect on three MDR cell lines (KB-C1, KB-V1 and KB-A1) selected with different agents, compared with their parental cell line (KB-3-1), there is no direct correlation between the level of multidrug resistance and the level of 2DG sensitivity. This contrasts with our previous finding that the three MDR KB cell lines KB-8-5, KB-8-5-11 and KB-C1, which were selected in progressively increasing concentrations of the same agent (colchicine), show progressively greater 2DG sensitivity with increasing levels of multidrug resistance (Bentley et al, 1996). The combination of these results suggests that, although Pgp level may contribute to the extent of 2DG-induced apoptosis in KB cell lines, different selecting agents select for other changes in MDR cells, distinct from those in Pgp expression, which influence the level of their sensitivity to 2DG.

Kaplan et al (1990) have shown that 2DG exposure leads to a reduction in ATP levels in MDR and sensitive cells, with a slightly greater reduction in the former. We have previously shown that a reduction in levels of the facilitative glucose transporter, GLUT-1, exists in MDR KB cells (Bentley et al, 1996), which may potentiate the reduction in ATP in MDR cells. ATP levels are thought to be crucial in signalling for apoptosis (Eguchi et al, 1997) and so depletion of ATP may be a contributory factor in the induction of the apoptotic pathway by 2DG in our cells. However, the lack of correlation between the GLUT-1 level and the extent of sensitivity to 2DG in different cell lines suggests that other mechanisms may also be involved.

It is possible to speculate on the nature of such mechanisms. Lavie et al (1996) have reported that there are higher levels of glucosylceramide in KB-V1 cells than their sensitive counterparts. Metabolites of such glycosphingolipids, such as ceramides, are suggested to have a second messenger function in some signal pathways regulating apoptosis (Hale et al, 1996; Hannun, 1996). Elevated levels of protein

© Cancer Research Campaign 1998

kinase C (PKC) have also been reported in KB-A1, KB-C1 and KB-V1 cells (Drew et al, 1994). PKC is known to be an important player in intracellular signalling leading to apoptosis, although its precise role is yet to be defined (Hale et al, 1996; Leszczynski, 1996).

In conclusion, we propose that a 2DG-stimulated apoptotic pathway differs in MDR KB cells from that in normal KB cells. Understanding changes in the pathway involved would provide the basis for rational suggestions of combinations of treatments to maximize the killing of MDR cells. Such studies could be of value in the clinical situation.

### ACKNOWLEDGEMENTS

We gratefully acknowledge financial support from the Medical Research Council and Yorkshire Cancer Research, valuable discussion with Jo Bentley and Mike Sharrard, and the excellent technical assistance of Ann Bamford.

### REFERENCES

Akiyama SI, Fojo A, Hanover JA, Pastan I and Gottesman MM (1985) Isolation and genetic characterisation of human KB cell lines resistant to multiple drugs. *Somatic Cell Mol Genet* **11**: 117–126

Bellamy WT and Dalton WS (1994) Multidrug resistance in the laboratory and clinic. *Adv Clin Chem* **31**: 1–61

Bentley J, Bell SE, Quinn DM, Kellett GL and Warr JR (1996) 2-Deoxy-D-glucose toxicity and transport in human multidrug resistant KB carcinoma cell lines. *Oncol Res* **8**: 77–84

Cohen JS and Lyon RC (1987) Multinuclear NMR study of the metabolism of drug-sensitive and drug-resistant human breast cancer cells. *Ann NY Acad Sci* **508**: 216–228

Drew L, Groome G, Hallam TJ, Warr JR and Rumsby MG (1994) Changes in protein kinase C subspecies expression and activity in a series of multidrug resistant human KB carcinoma cell lines. *Oncol Res* **6**: 429–438

Eguchi Y, Shimizu S and Tsujimoto Y (1997) Intracellular ATP levels determine cell death fate by apoptosis or necrosis. *Cancer Res* **57**: 1835–1840

Fanciulli M, Bruno T, Castiglione S, Del Carlo C, Paggi MG and Floridi A (1993) Glucose metabolism in adriamycin-sensitive and -resistant LoVo human colon carcinoma cells. *Oncol Res* **5**: 357–362

Filipits M, Suchomel RW, Zöchbauer S, Malayeri R and Pirker R (1996) Clinical relevance of drug resistance genes in malignant diseases. *Leukemia* **10**: S10–S17

Germann UA (1996) P-glycoprotein – a mediator of multidrug resistance in tumour cells. *Eur J Cancer* **32A**: 927–944

Gottesman MM and Pastan I (1993) Biochemistry of multidrug resistance mediated by the multidrug transporter. *Annu Rev Biochem* **62**: 385–427

Hale AJ, Smith CA, Sutherland LC, Stoneman VEA, Longthorne VL, Culhane AC and Williams GT (1996) Apoptosis: molecular regulation of cell death. *Eur J Biochem* **236**: 1–26

Hannun YA (1996) Functions of ceramide in coordinating cellular responses to stress. *Science* **274**: 1855–1859

Harrison SA, Buxton JM, Clancy BM and Czech MP (1990) Insulin regulation of hexose transport in mouse 3T3-L1 cells expressing the human HepG2 glucose transporter. *J Biol Chem* **265**: 20106–20116

Jain VK, Kalia VK, Sharma R, Maharajan V and Menon M (1985) Effects of 2-deoxy-D-glucose on glycolysis, proliferation kinetics and radiation response of human cancer cells. *Int J Radiat Oncol Biol Phys* **11**: 943–950

Kaplan O, Navon G, Lyon RC, Faustino PJ, Straka EJ and Cohen JS (1990) Effects of 2-deoxyglucose on drug-sensitive and drug-resistant human breast cancer cells: toxicity and magnetic resonance spectroscopy studies of metabolism. *Cancer Res* **50**: 544–551

Kaplan O, Jaroszewski JW, Clarke R, Fairchild CR, Schoenlein P, Goldenburg S, Gottesman MM and Cohen JS (1991) The multidrug resistance phenotype: $^{31}P$ nuclear magnetic resonance characterisation and 2-deoxyglucose toxicity. *Cancer Res* **51**: 1638–1644

Kaufmann SH, Desnoyers S, Ottaviano Y, Davidson NE and Poirier GG (1993) Specific cleavage of poly(ADP-ribose) polymerase: an early marker of chemotherapy-induced apoptosis. *Cancer Res* **53**: 3976–3985

Koopman G, Reutelingsperger CPM, Kuijten GAM, Keehnen RMJ, Pals ST and van Oers MHJ (1994) Annexin V for flow cytometric detection of phosphatidylserine expression on B cells undergoing apoptosis. *Blood* **84**: 1415–1420

Lavie Y, Cao H, Bursten SL, Giuliano AE and Cabot MC (1996) Accumulation of glucosylceramides in multidrug-resistant cancer cells. *J Biol Chem* **271**: 19530–19536

Leszczynski D (1996) The role of protein kinase C in regulation of apoptosis: a brief overview of the controversy. *Cancer J* **9**: 308–313

Lyon RC, Cohen JS, Faustino PJ, Megnin F and Myers CE (1988) Glucose metabolism in drug-sensitive and drug-resistant human breast cancer cells monitored by magnetic resonance spectroscopy. *Cancer Res* **48**: 870–877

Malayeri R, Filipits M, Suchomel RW, Zöchbauer S, Lechner K and Pirker R (1996) Multidrug resistance in leukemias and its reversal. *Leukemia Lymphoma* **23**: 451–458

Martin SJ and Cotter TG (1991) Ultraviolet B irradiation of human leukaemia HL-60 cells *in vitro* induces apoptosis. *Int J Radiat Biol* **59**: 1001–1016

Martin SJ, Reutelingsperger CPM, McGahon AJ, Rader JA, van Schie RCAA, LaFace DM and Green DR (1995) Early redistribution of plasma membrane phosphatidylserine is a general feature of apoptosis regardless of initiating stimulus: inhibition by overexpression of Bcl-2 and Abl. *J Exp Med* **182**: 1545–1556

Rasmussen J, Hansen LL, Friche E and Jaroszewski JW (1993) $^{31}P$ and $^{13}C$ NMR spectroscopic study of wild type and multidrug resistant Ehrlich ascites tumour cells. *Oncol Res* **5**: 119–126

Shen DW, Cardarelli C, Hwang J, Cornwell M, Richert N, Ishiii S, Pastan I and Gottesman MM (1986) Multiple drug resistant human KB carcinoma cells independently selected for high level resistance to colchicine, adriamycin or vinblastine show changes in expression of specific proteins. *J Biol Chem* **261**: 7762–7770

Shustik C, Dalton W and Gros P (1995) P-glycoprotein-mediated multidrug resistance in tumour cells: biochemistry, clinical relevance and modulation. *Mol Aspects Med* **16**: 1–78

Trock BJ, Leonessa F and Clarke R (1997) Multidrug resistance in breast cancer: a meta-analysis of MDR1/gp170 expression and its possible functional significance. *J Natl Cancer Inst* **89**: 917–931

Umansky SR (1996) Apoptosis: molecular and cellular mechanisms (a review). *Mol Biol* **30**: 285–295

van Engeland M, Ramaekers FCS, Schutte B and Reutelingsperger CPM (1996) A novel assay to measure loss of plasma membrane asymmetry during apoptosis of adherent cells in culture. *Cytometry* **24**: 131–139

Wick AN, Drury DR, Nakada HI and Wolfe JB (1957) Localisation of the primary metabolic block produced by 2-deoxyglucose. *J Biol Chem* **224**: 963–969

 © Cancer Research Campaign 1998